# Safety and Efficacy of Mosunetuzumab: Experience in the Hospital Cardinale Giovanni Panico

**DOI:** 10.3390/antib15030040

**Published:** 2026-05-13

**Authors:** Giulio Turco, Donatella Tarantino, Antonietta Giuseppa Ferraro, Giuseppina Greco, Domenico Tricarico

**Affiliations:** 1 Operative Complex Unit of Hospital Pharmacy Pia Fondazione di Culto e Religione Card. G. Panico, 73039 Tricase, Italy; g.turco@piafondazionepanico.it (G.T.); d.tarantino@piafondazionepanico.it (D.T.); gferraro@piafondazionepanico.it (A.G.F.); 2 Operative Complex Unit of Hematology Pia Fondazione di Culto e Religione Card. G. Panico, 73039 Tricase, Italy; hos@piafondazionepanico.it; 3 Hospital School of Pharmacy, Department of Pharmacy, Pharmaceutical Sciences, University of Bari Aldo Moro, 70125 Bari, Italy

**Keywords:** follicular lymphoma, mosunetuzumab, bispecific antibodies, CD20, CD3, immunotherapy, relapsed lymphoma

## Abstract

Background/Objective: Follicular lymphoma (FL) is one of the most common indolent B-cell non-Hodgkin lymphomas (NHL) and is characterized by recurrent relapses despite advances in therapy. Bispecific antibodies that redirect T lymphocytes toward malignant B cells represent a major innovation in the treatment of relapsed or refractory disease. Mosunetuzumab is a CD20×CD3 bispecific antibody that induces T-cell mediated cytotoxicity against B-cell malignancies. In this manuscript, we describe the clinical experience with mosunetuzumab in three patients with relapsed or refractory FL treated at the Hospital Card. G. Panico, Tricase (LE). Methods: Clinical history, prior therapies, treatment responses, and safety outcomes are reported. Results: The cases illustrate the potential efficacy and manageable safety profile of mosunetuzumab in heavily pretreated FL patients. Conclusion: The effectiveness of this drug is confirmed in our center.

## 1. Introduction

Follicular lymphoma (FL) represents an indolent B-cell malignancy originating from germinal center lymphocytes. Over the last decades, the therapeutic landscape has significantly improved patient survival; however, FL remains a chronic and generally incurable disease characterized by repeated relapses and biological heterogeneity [1]. Some patients experience early disease progression, histologic transformation, or toxicity associated with treatment strategies [1]. Current first- and second-line therapies mainly include chemo-immunotherapy regimens such as bendamustine plus rituximab (BR), bendamustine plus obinutuzumab (BO), and the combination of cyclophosphamide, doxorubicin, vincristine, and prednisone (CHOP) with anti-CD20 monoclonal antibodies. In selected cases, autologous stem cell transplantation (SCT) may represent an additional therapeutic option, particularly in patients experiencing early treatment failure [2].

Despite the availability of multiple therapeutic approaches, FL typically follows a relapsing clinical course, and disease control intervals frequently become shorter with each subsequent line of therapy. Consequently, new treatment modalities with improved efficacy and manageable toxicity are needed. Among emerging immunotherapeutic strategies, bispecific antibodies (*bsAbs*) have gained considerable attention because they simultaneously target tumor cells and immune effector cells, thereby promoting targeted immune-mediated cytotoxicity.

### 1.1. Follicular Lymphoma

Follicular lymphoma is the second most frequent subtype of non-Hodgkin lymphoma (NHL) and is characterized by a neoplastic proliferation of germinal center B cells [3]. The development of FL involves a complex and multistep process that includes genetic and epigenetic alterations occurring in B lymphocytes. Tumor tissues typically contain a mixture of small cleaved cells (centrocytes) and larger blastoid cells (Centro blasts), which resemble the cellular composition of normal germinal centers [4].

**Figure 1:**
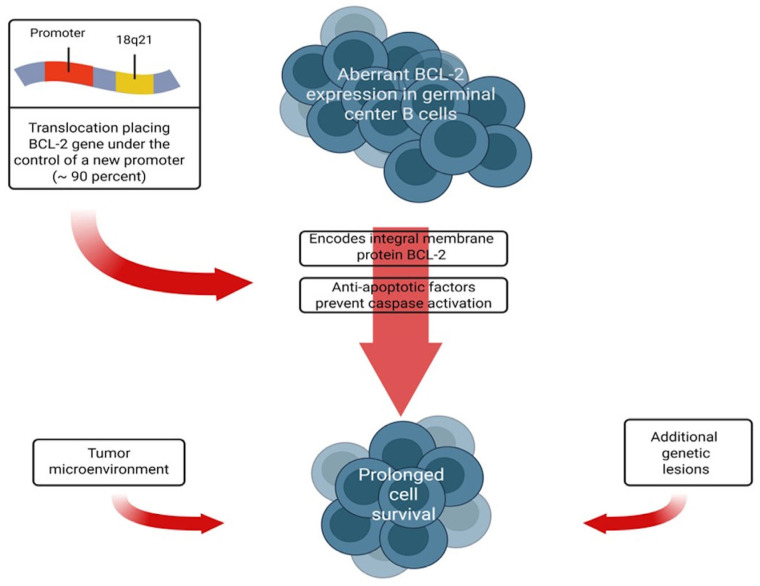
The follicular lymphoma (*FL*) leads to the replication of a malignant B-cell clone of germinal origin. Most *FL* tumors have translocations or mutations that result in increased expression of the *BCL-2* gene. Approximately 85% of cases have a translocation between the long arm of chromosome 18 and chromosome 14 on the immunoglobulin heavy chain gene resulting in *t(14;18) (q32; q21).* Overexpression of the anti-apoptotic factor BCL-2 is not sufficient to cause *FL* and other factors, such as the tumor microenvironment and other genetic lesions, probably contribute to the pathogenesis.

A hallmark molecular event in FL is the chromosomal translocation *t(14;18)(q32;q21)*, which results in overexpression of the anti-apoptotic protein BCL-2 [5]. Although *BCL-2* upregulation contributes to prolonged cell survival, this alteration alone is insufficient for malignant transformation. Additional genetic lesions and interactions with the tumor microenvironment are required for disease development and progression [6,7]. Recent genomic studies have highlighted the role of immune cells, stromal components, and cytokine networks in shaping tumor growth and clinical outcome [8,9,10,11,12,13] (Figure 1).

Clinically, FL often presents with painless lymphadenopathy involving cervical, axillary, or inguinal lymph node regions [14,15,16]. Many patients remain asymptomatic at diagnosis, although a minority present with systemic ‘B symptoms’ such as fever, night sweats, and weight loss [17]. Extranodal involvement may occur but is less common, while bone marrow infiltration is frequently observed during staging procedures [18,19,20,21,22,23,24]. The diagnosis of FL is generally established through excisional lymph node biopsy, which allows histological assessment of follicular architecture and cellular morphology. Immunophenotypic analysis and molecular studies may support the diagnosis and help distinguish FL from other lymphoid malignancies [14]. Some infantile FL are often negative to *BCL2* [25,26,27].

Immunophenotypic analysis and molecular studies may support the diagnosis and help distinguish FL from other lymphoid malignancies [14]. Several patients show no signs of FL at further evaluation [28,29]. Duodenal-type FL is recognized as a distinct entity confined to the gastrointestinal tract—most frequently the duodenum—and commonly appears as multiple small polyps [30]. Although rare [31,32], it is typically limited to the mucosa, often retains at least a partial follicular growth pattern, and usually displays grade 1–2 cytology [31].

The clinical course of FL is heterogeneous. Some patients experience a prolonged, intermittently active disease trajectory and may remain without treatment for five years or longer [33]. Others present with more extensive involvement and faster growth kinetics, leading to symptoms or organ compromise (e.g., pain, obstruction, or dysfunction) that necessitate therapy [34]. While early studies suggest that certain pathogenic mutations acquired in FL could carry prognostic information, additional evidence is required before routine genome sequencing can be broadly incorporated into standard care pathways [14].

Grading systems differ slightly across classification frameworks. The International Consensus Classification (ICC) grades FL on a 1–3 scale [27], whereas the WHO scheme consolidates disease into classical FL (corresponding to ICC grades 1–3a) and follicular large B-cell lymphoma (ICC grade 3b) [27]. Grade may have prognostic relevance—particularly grade 3b, which is generally considered to follow a more aggressive clinical course compared with other FL grades [35,36].

Comprehensive pretreatment evaluation is required to define disease extent in Stages I–IV, and to capture comorbidities that may influence therapy selection. Beyond history and physical examination, assessment includes review of pathology to confirm diagnostic adequacy, laboratory testing [37,38], and baseline imaging. Imaging may be performed with contrast-enhanced CT or FDG PET/CT, which can help map anatomic involvement and metabolic activity [39,40]. The urgency to initiate therapy varies and should be individualized based on symptoms, organ function, and available options.

### 1.2. Initial Treatment

Strategy is guided by stage, grade, and distribution of disease. For Stage I FL (grades 1–3a), involved-site radiotherapy with curative intent is generally favored when all sites can be encompassed with acceptable toxicity; if radiotherapy is not feasible, an initial observation approach may be preferred [37,38]. For Stage I grade 3b disease, regimens used for clinically aggressive lymphomas (such as diffuse large B-cell lymphoma) are typically recommended [37,38]. Stage II (grades 1–3a) is often managed similarly to advanced disease, although selected patients may still be offered radiotherapy depending on clinical context [37,38]. Only a minority of FL cases present as Stage I–II (approximately 15–30%) [41,42]. At diagnosis, most patients have Stage II (approximately 11–16%), Stage III (22–33%), or Stage IV disease (26–40%) [43,44]. Management of Stage III–IV FL depends on grade, symptoms/organ dysfunction, tumor burden, and disease kinetics [37,38].

For asymptomatic, low-tumor-burden Stage II–IV FL, single-agent rituximab can be used as an alternative to a watch-and-wait strategy when patients prefer early treatment. Regimens evaluated in randomized studies include four weekly doses (e.g., days 1, 8, 15, 22) or four weekly doses followed by maintenance administration every two months for two years [45,46].

Anti-CD20 immunotherapy (rituximab or obinutuzumab) is a cornerstone for symptomatic FL [47,48]. Common options—ordered from lower to higher intensity—include: (i) rituximab as a defined course; (ii) rituximab plus chemotherapy for 6–8 cycles; and (iii) immunochemotherapy followed by maintenance anti-CD20 therapy for two years. In practice, rituximab-chemotherapy is frequently chosen over rituximab alone because indirect evidence supports a faster and deeper response. Nevertheless, single-agent rituximab remains appropriate for patients with significant comorbidities, lower tumor burden, and/or slowly progressive disease where chemotherapy is undesirable [37,38].

Multiple chemotherapy backbones have been combined with rituximab or obinutuzumab. Bendamustine-rituximab (BR) is commonly preferred because of a favorable toxicity profile and evidence of comparable efficacy to R-CHOP in several studies [37,38]. R-CVP is an alternative but may yield lower response rates. Obinutuzumab can be combined with bendamustine, CHOP, or CVP and followed by maintenance obinutuzumab; in one study, this approach improved progression-free survival but increased toxicity and costs [49]. Fludarabine-based regimens are generally avoided due to high toxicity [50,51]. Lenalidomide-based combinations (e.g., lenalidomide with rituximab or obinutuzumab) are increasingly used, particularly in the relapsed/refractory setting [37,38,49,50,51].

For Relapsed or Refractory Follicular Lymphoma, before initiating therapy for suspected relapse, repeat biopsy is recommended with a low threshold to confirm recurrence and to evaluate for histologic transformation. FDG PET/CT can establish a new baseline and guide biopsy toward the most metabolically active site, which is important because treatment strategy and prognosis differ substantially when transformation is present [51,52].

As with frontline disease, asymptomatic relapse does not necessarily require immediate therapy, but close surveillance is warranted. In general, the same clinical triggers used for initial treatment—symptoms, cytopenias, threatened organ function, or rapid progression—also apply at recurrence or progression [52].

Many patients who relapse more than 24 months after initial immunochemotherapy (or more than 12 months after single-agent rituximab) can anticipate long survival with intermittent treatment, often approaching that of the general population [53]. While cure is uncommon, contemporary therapies frequently achieve complete or partial remissions. The goals of care include symptom control, reversal of cytopenias, and improvement in quality of life. For later relapses, re-treatment with anti-CD20 therapy (rituximab or obinutuzumab)—alone or combined with agents such as lenalidomide—is often favored over repeated intensive immunochemotherapy, although chemotherapy-based options remain acceptable for selected patients when a time-limited intensive strategy is preferred [52,53].

### 1.3. Bispecific Antibodies

**Figure 2:**
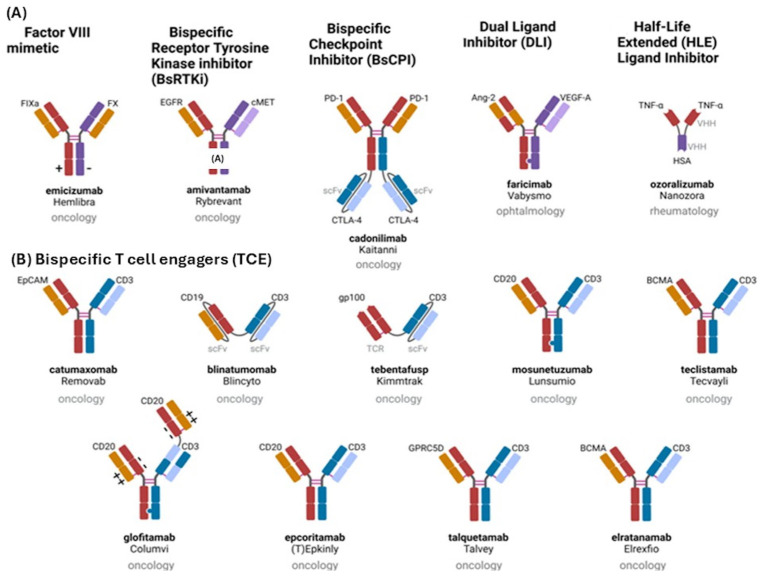
Molecular structure of novel engineered antibodies: (**A**) mimetic of factor VIII, inhibition of dual signaling, bispecific receptor tyrosine kinase (*RTK*) inhibitor (*BsRTI*), bispecific checkpoint inhibitor (*BsCPI*), double ligand inhibitor (*DLI*), extended half-life ligand inhibitor (*HLE*). (**B**) T cell engager (*TCE*). The symbols + and – indicates the orientation of the interaction sites of the CD3 on the CD20 with ++ head and – – tail, respectively.

These are engineered molecules capable of simultaneously binding two different antigens or epitopes [54,55,56,57]. In oncology, these agents often link tumor cells with immune effector cells, thereby facilitating targeted immune activation and tumor cell destruction and not target immune reaction. Several structural formats have been developed, including IgG-like and non-IgG-like antibodies, each with specific pharmacological characteristics and clinical advantages (Figure 2) [57,58,59,60].

Although generally manageable, treatment with bispecific antibodies may be associated with immune-related adverse events such as cytokine release syndrome (CRS), immune effector cell-associated neurotoxicity syndrome (ICANS), infusion-related reactions, and opportunistic infections [61,62]. Careful patient monitoring and step-up dosing strategies are therefore important to minimize treatment-related toxicity. The infusion-related reactions are commonly observed with different mAbs, are structure related and have economic impact [63,64,65]. Mosunetuzumab is a CD20×CD3 bispecific antibody designed to redirect T lymphocytes toward CD20-expressing malignant B cells. Through this mechanism, the drug induces T-cell activation and subsequent cytotoxic elimination of lymphoma cells [66,67]. Clinical trials have demonstrated promising efficacy in patients with relapsed or refractory FL who previously received multiple lines of therapy [58,59]. These advances have positioned mosunetuzumab as an important therapeutic option in relapsed or refractory FL, particularly for patients who are not candidates for more intensive treatments such as CAR-T cell therapy. The structure and function of bivalent antibodies are reported in Figure 2 [54,55,56,57,68,69,70].

**Figure 3:**
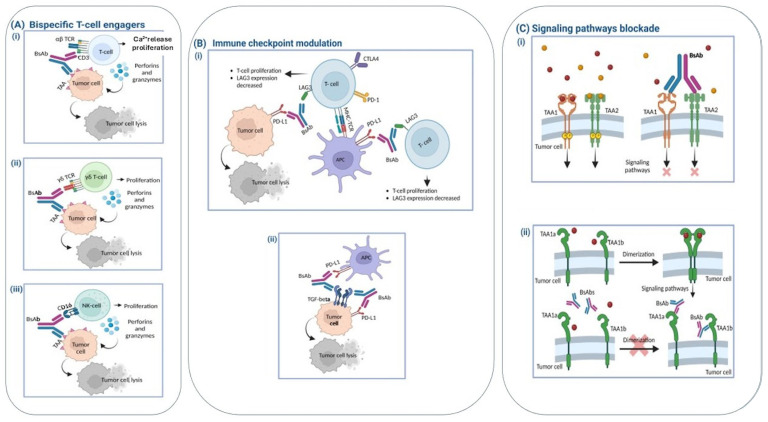
(**A**) Bispecific immune cell engagers. (**i**) Bispecific antibodies that bind both the CD3 subunit of the T-cell receptor (TCR) and a selected tumor-associated antigen (TAA) promote the formation of an immune synapse between T cells and tumor cells. This interaction causes calcium release mediated by CRAC channels that activates T cells, which release perforins and granzymes that induce tumor cell lysis. (**ii**) Bispecific γδ T-cell engagers simultaneously target Vγ9Vδ2 T-cell receptors and a specific TAA, leading to activation of γδ T cells and cytotoxic killing of tumor cells. (**iii**) Bispecific natural killer (NK) cell engagers bind CD16 receptors on NK cells together with a TAA on tumor cells, stimulating NK-cell activation and the release of cytotoxic granules that destroy tumor cells. (**B**) Immune checkpoint modulation. (**i**) Immune checkpoint–blocking *bsAbs* can simultaneously bind lymphocyte activation gene-3 (LAG-3) on T cells and programmed death ligand-1 (PD-L1) on tumor cells or antigen-presenting cells (APCs). This dual blockade enhances T-cell activation, promoting the release of perforins and granzymes that mediate tumor cell killing while reducing inhibitory signaling. (**ii**) Some *bsAbs* simultaneously target immune checkpoint molecules together with proteins involved in other signaling pathways, thereby integrating immune modulation with direct antitumor signaling inhibition. (**C**) Signal pathway blockade (X). (**i**) Certain *bsAbs* concurrently bind epidermal growth factor receptor (EGFR) and c-MET, inhibiting ligand-induced phosphorylation, suppressing downstream signaling pathways, and promoting receptor degradation. (**ii**) Biparatopic *bsAbs* can bind two distinct epitopes on the same target molecule, enhancing the inhibition of receptor activity. Abbreviations: APC, antigen-presenting cell; CD, cluster of differentiation; CTLA-4, cytotoxic T-lymphocyte antigen-4; MHC, major histocompatibility complex; PD-1, programmed death-1. Figure adapted from [70].

The therapeutic activity of bispecific antibodies (*bsAbs*) is largely determined by their biological targets, which define their mechanism of action. Based on their functional properties, *bsAbs* can be broadly categorized into three principal classes (Figure 3A–C).

#### 1.3.1. Immune Cell Engagers

Bispecific T-cell engagers (TCEs) are engineered to physically connect endogenous CD4^+^ and CD8^+^ T lymphocytes with tumor cells. This occurs through the simultaneous binding of the CD3ε subunit of the T-cell receptor (TCR) complex and a specific tumor-associated antigen (TAA) expressed on malignant cells [68,69,70]. The resulting immune synapse triggers T-cell activation and leads to the release of cytotoxic mediators and pro-inflammatory cytokines that promote tumor cell destruction [71,72,73,74,75]. This action is mediated by ion channels more specifically calcium release activated channel (CRAC). Indeed, mosunetuzumab activates CD3 signaling increasing short lived calcium release via IP_3_ on endoplasmic reticulum (ER), activation of calcium channel CRAC with sustained long lasting Ca^2+^ entry and calcium dependent NFAT transcription with T-cell activation and cytotoxicity [76,77,78,79,80] (Figure 3A). 

Immune checkpoint modulation: Another group of *bsAbs* is designed to target immune checkpoint pathways. Dual immune checkpoint–blocking *bsAbs* simultaneously bind inhibitory receptors—such as PD-1, CTLA-4, LAG-3, or TIGIT—on the T-cell surface, while the second binding arm interacts with another checkpoint molecule expressed on T cells, tumor cells, or antigen-presenting cells [71,81,82]. By interfering with these inhibitory signals, these *bsAbs* enhance T-cell activation and restore antitumor immune responses.

#### 1.3.2. Inhibition of Signaling Pathways

*BsAbs* can also disrupt oncogenic signaling networks by simultaneously targeting two different antigens or distinct epitopes of the same receptor involved in tumor growth pathways. Dual targeting of driver signaling cascades can improve the therapeutic efficacy of *bsAbs* [83] and may reduce the development of drug resistance in combined therapy [74].

Several mechanisms underlie the ability of *bsAbs* to interfere with signaling pathways. The primary mechanism involves blocking receptor–ligand interactions, thereby preventing activation of downstream signaling cascades. In addition, *bsAbs* may induce receptor internalization, limiting receptor cross-linking and dimerization (both homo- and heterodimerization). These effects can ultimately suppress processes such as angiogenesis and tumor cell proliferation. Furthermore, IgG-based *bsAbs* that retain an Fc domain can activate immune effector mechanisms including antibody-dependent cellular phagocytosis (ADCP) and antibody-dependent cellular cytotoxicity (ADCC), which contribute to apoptosis of tumor cells [72,73].

Importantly, *bsAbs* may also help overcome resistance to tyrosine kinase inhibitors (TKIs) [84,85]. Resistance can arise through the up-regulation of compensatory signaling pathways, such as activation of the mesenchymal-epithelial transition (MET) pathway observed in epidermal growth factor receptor (EGFR)–mutated non-small cell lung cancer (NSCLC) [74]. 

Adverse reactions under monitoring are Cytokine Release Syndrome (CRS), immune effector cell-associated neurotoxicity syndrome (ICANS Immune effector cell-associated neurotoxicity syndrome) that is associated with T cells immunoactivities and BBB disruption and infusion-related reactions: (IRRs infusion-related reactions) that can be fatal [86,87,88,89,90,91,92,93]. Opportunistic infections of respiratory track due to gram-negative bacterial and fungal (e.g., *Aspergillus* spp.), and viral (e.g., cytomegalovirus) infections also occur [93].

Despite the efficacy of monesetuzumab being well established, the approval of mosunetuzumab was based on the results of an international, multicenter, phase II study of 90 patients with *FL RR* (relapsed refractory follicular lymphoma) after at least two previous lines of systemic therapy [58,59,94]. Mosunetuzumab targeting CD3 and CD20, is approved for patients with *FL R/R* who have received ≥2 previous lines of treatment [58,59,95,96]. The effectiveness remains to be evaluated in specific contexts.

Insufficient patient care at the Center and the Adverse Drug Reaction (ADR) of this drug can limit the effectiveness of the therapy and require monitoring. In this manuscript we evaluated the effectiveness of mosunetuzumab in our center.

## 2. Methods

In the A.O. Pia Fondazione di Culto e Religione ‘Card. G. Panico’ in Tricase, (Hospital Card. G. Panico) in the province of Lecce (LE), bispecific antibody therapies have been in use for three years. In particular, *bsAb* mosunetuzumab, first launched as compassionate use programmed in May 2022 approved at Local Ethical Committee (CEL) of I.R.C.C.S., Oncology Hospital, Giovanni Paolo II Bari, and still in use today as a *reimbursable* drug authorized by *AIFA*, was approved for the treatment of eight patients in the Hospital Card. G. Panico.

**Figure 4:**
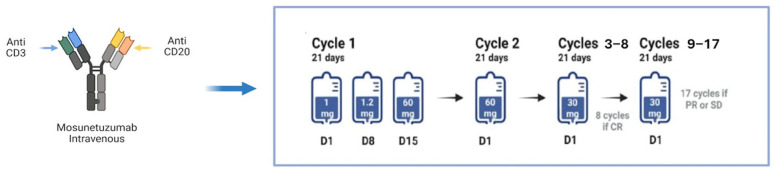
Mosunetuzumab doses and schedule of administration.

Mosunetuzumab is administered intravenously (EV) in 21-day cycles, with cycle 1 consisting of a stepwise dosage: 1 mg on day 1 of cycle 1, 2 mg on day 8 of cycle 1, 60 mg on day 15 of cycle 1, 60 mg on day 1 of cycle 2, 30 mg on day 1 of cycle 3 and thereafter. Treatment is stopped after cycle 8 for patients with complete response (CR), while patients with partial response (PR) or stable disease (SD) continue treatment for up to 17 cycles (Figure 4) [58,59].

Evaluation of adverse reactions and radiotherapy treatments were based on previous data and protocols [97,98,99,100,101,102,103,104,105,106]. 

## 3. Results: Experience in the Hospital Card. G. Panico, Patient Cases

### 3.1. Patient Cases

Number of patients with relapsed or refractory FL treated with the drug mosunetuzumab, from 2022 to date, in the Hospital Card. G. Panico is eight. Six patients have completed treatment with mosunetuzumab, one patient is being treated with mosunetuzumab, and one patient has changed treatment.

Below is the clinical history of three patients who completed treatment with mosunetuzumab.

#### 3.1.1. Patient 1

Patient 1 was an aged man with hypertension, benign prostatic hyperplasia, chronic obstructive pulmonary disease, a remote smoking history, and a central venous access device. He had previously been treated for B-cell non-Hodgkin lymphoma many years earlier with six cycles of R-CHOP. At relapse, he was diagnosed at our center with follicular lymphoma, stage IIA with bulky abdominal disease (FLIPI-2 score 2; GELF score 2).

Restaging PET/CT at relapse showed metabolically active disease in the left iliac-obturator region and an additional hypermetabolic focus adjacent to the sigmoid colon. Abdominal lymph node biopsy confirmed follicular lymphoma. The patient then received six cycles of bendamustine plus rituximab as second-line therapy and tolerated treatment well, with preserved performance status and no major complications.

Post-treatment PET/CT demonstrated persistent metabolically active disease in the left iliac-obturator region, consistent with refractory lymphoma. Because the disease was refractory to two prior lines of therapy (R-CHOP and bendamustine-rituximab), the patient started mosunetuzumab with standard step-up dosing administered under in-patient observation during cycle 1.

Mosunetuzumab was given for eight cycles, after which treatment was stopped according to protocol because complete response had been achieved. During the first cycle, the patient developed myalgias; pruritic rash involving the axillary, submental, lumbar, and inguinal regions; and fever up to 38.8 °C. These events were consistent with cytokine release syndrome and resolved with supportive management. No subsequent serious toxicities were observed.

End-of-treatment PET/CT showed complete metabolic response, with interval reduction of residual soft tissue along the left iliac vessels and no new sites of disease. Follow-up imaging performed approximately six months after completion of treatment confirmed an ongoing complete remission.

#### 3.1.2. Patient 2

Patient 2 was an adult man with a remote smoking history, congenital solitary kidney, and a central venous access device. He was initially diagnosed with grade 3A, stage IV follicular lymphoma (FLIPI-2 score 2).

Front-line treatment consisted of six cycles of R-CHOP, which produced a marked reduction in nodal disease and complete remission, followed by rituximab maintenance. Approximately three years later, biopsy and PET/CT confirmed relapse as grade 2, stage IVa follicular lymphoma. He then received second-line R-DHAOX with dose reduction of oxaliplatin because of his solitary kidney, followed by autologous stem cell transplantation, and again achieved complete remission.

Approximately 2.5 years after transplantation, PET/CT and biopsy documented a further relapse with metabolically active nodal and soft-tissue disease. Because the lymphoma was refractory to two prior lines of therapy, including transplant-based treatment, the patient started mosunetuzumab with standard step-up dosing and in-patient monitoring during cycle 1.

He completed eight cycles of mosunetuzumab and maintained excellent performance status, with normal hematologic, liver, and kidney function throughout treatment. A mild grade 1 cytokine release syndrome occurred after the first administration and resolved without sequelae.

**Figure 5:**
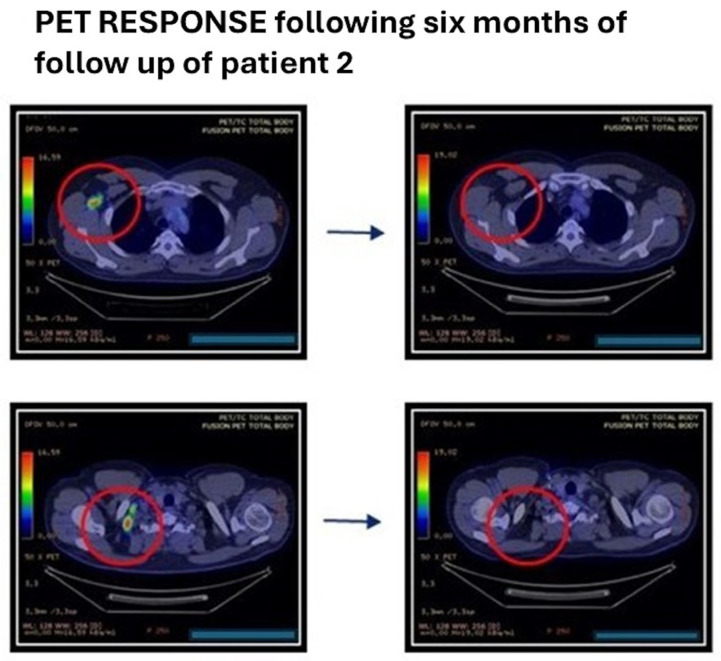
Differences *Pet* patient 2 before treatment with mosunetuzumab and after four cycles of mosunetuzumab therapy. Red circle indicates the tumor area.

Interim PET/CT after four cycles showed a marked reduction in previously involved cervical and inguinal-femoral lymph nodes, and end-of-treatment PET/CT after eight cycles confirmed complete metabolic response. Follow-up clinical and imaging assessments at approximately 6, 8, and 12 months after treatment completion continued to show complete remission **(**Figure 5).

#### 3.1.3. Patient 3

Patient 3 was an adult man with anxiety disorder, prior severe hypersensitivity reaction to pegfilgrastim, previous SARS-CoV-2 infection, and a central venous access device. He had grade 2, stage IVa follicular lymphoma (FLIPI-1 score 2; FLIPI-2 score 1).

He received first-line O-CHOP and had persistent disease on post-treatment PET/CT. Second-line salvage therapy with R-DHAP followed by DHAOX was then administered; however, repeat PET/CT showed progressive metabolically active pelvic-abdominal disease. Third-line treatment with rituximab plus lenalidomide was subsequently started; lenalidomide required temporary interruptions because of pruritus and papillomatous skin lesions, which later resolved. Reassessment after this regimen showed stable disease.

Because the lymphoma remained refractory after three prior lines of therapy, the patient started mosunetuzumab with standard step-up dosing and inpatient monitoring during cycle 1.

He completed eight cycles with good general condition, normal cardiopulmonary examination, and no evidence of cytokine release syndrome or immune effector cell-associated neurotoxicity syndrome. Occasional headaches during the first cycle were consistent with his prior history and were not considered treatment related.

**Figure 6:**
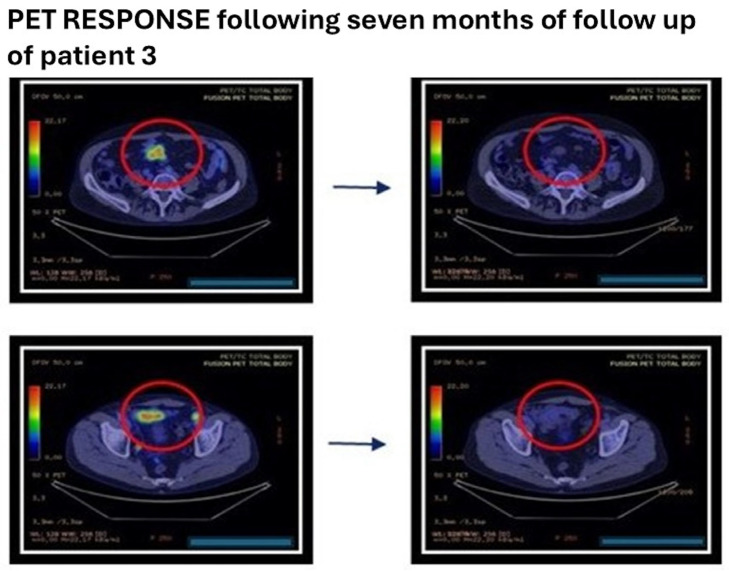
Differences *patient 3* before treatment with mosunetuzumab and after eight cycles of mosunetuzumab therapy. Red circle indicates the tumor area.

Interim PET/CT after four cycles showed a marked reduction in mesenteric, mesogastric, paravesical, retro vesical, presacral, obturator, and iliac disease sites. End-of-treatment PET/CT after eight cycles demonstrated near-complete disappearance of residual uptake, consistent with complete metabolic response. Follow-up clinical and imaging assessments performed approximately 7, 11, and 13 months after treatment completion confirmed ongoing complete remission (Figure 6).

## 4. Conclusions

Bispecific antibodies such as mosunetuzumab, a full-length IgG1 CD20×CD3 antibody that redirects T cells against malignant B cells, are expanding the treatment options for relapsed or refractory follicular lymphoma. In this case series, all three heavily pretreated patients achieved complete remission, with durable responses during follow-up and no unexpected safety signals.

The comparative analysis of the GO29781 study shows that the three patients described here meet the eligibility and inclusion criteria established for this single-arm, multi-center, phase I/II clinical trial for the drug mosunetuzumab, which was conducted in 35 countries around the world between 2019 and 2020 prior to its market launch [58,59,94]. The eligibility criteria included being aged 18 years or over, having histologically confirmed follicular lymphoma (grade 1–3a), and having relapsed or not responded to two or more prior lines of treatment, including anti-CD20 therapy and an alkylating agent (Table 1); however, no Italian patients were recruited at that time.

In these three cases, the clinical outcomes were consistent with the primary and secondary endpoints reported in the GO29781 study, with sustained responses, acceptable tolerability, and ongoing remission during follow-up.

## Figures and Tables

**Table 1:** Case-Study Comparison.

	GO29781 Single-Arm, Multicenter, Phase II Study	Patient 1	Patient 2	Patient 3
Grade FL	FL (Gr 1–3a)	FL (Gr 2a)	FL (Gr 3a)	FL (Gr 2a)
Age Patients	>18 years	75 years	48 years	61 years
Prior Regimens	FL R/R to ≥2 prior regimens ≥1 anti-CD20 antibody ≥1 alkylating agent	R-CHOP schedule R-BENDA schedule	R-CHOP schedule R-DHAOX schedule FEAM schedule ASCT	O-CHOP schedule R-DHAP schedule DHAOX schedule Rituximab and lenalidomide
Therapy Cycles	8 cycles if CR after cycles 8 17 cycles if PR/SD after cycles 8	8 cycles	8 cycles	8 cycles
Dosage	Step-up dosing	Step-up dosing	Step-up dosing	Step-up dosing
Primary Endpoints	Complete Response Rate	Complete Response Rate	Complete Response Rate	Complete Response Rate
Secondary Endpoints	Partial Response Rate–Progression Free Survival	Progression Free Survival	Progression Free Survival	Progression Free Survival
Toxicity/Adverse Reactions	CRS/ICANS	Lower CRS	Lower CRS	No CRS/ICANS

Abbreviations: FL, NHL; R/R, relapsed or refractory FL; R-CHOP SCHEDULE, rituximab, cyclophosphamide, doxorubicin, vincristine, prednisone; R-BENDA SCHEDULE, rituximab, bendamustine; R-DHAOX SCHEDULE, rituximab, dexamethasone, cytarabine, oxaliplatin; FEAM SCHEDULE, fotemustine, cytarabine, etoposide, melphalan; ASCT, autologous stem cell transplantation in candidate patients; O-CHOP SCHEDULE, obinutuzumab, cyclophosphamide, doxorubicin, vincristine, prednisone; R-DHAP SCHEDULE rituximab, dexamethasone, cytarabine and cisplatin; DHAOX SCHEDULE, dexamethasone, cytarabine, oxaliplatin; CR, complete response; PR, partial response; SD, stable disease; CRS, cytokine release syndrome; ICANS, immune effector cell-associated neurotoxicity syndrome.

## Data Availability

The data presented in this study are available upon request from the corresponding author due to limited patients permission for diffusion.
